# Recent Advances in Fluorescent Nanoprobes for Food Safety Detection

**DOI:** 10.3390/molecules28145604

**Published:** 2023-07-24

**Authors:** Huanxiang Yuan, Yutong Li, Jiaqi Lv, Yunhe An, Di Guan, Jia Liu, Chenxiao Tu, Xiaoyu Wang, Huijuan Zhou

**Affiliations:** 1 Department of Chemistry, College of Chemistry and Materials Engineering, Beijing Technology and Business University, Beijing 100048, China; 2 Institute of Analysis and Testing, Beijing Academy of Science and Technology (Beijing Center for Physical & Chemical Analysis), Beijing 100089, China; 3 Food Science and Engineering College, Beijing University of Agriculture, Beijing 102206, China; 4 School of Materials Science and Engineering, University of Science and Technology Beijing, Beijing 100083, China

**Keywords:** fluorescent nanoprobes, residues, food safety, limit of detection, recovery rate

## Abstract

Fluorescent nanoprobes show similar fluorescence properties to traditional organic dyes, but the addition of nanotechnology accurately controls the size, shape, chemical composition, and surface chemistry of the nanoprobes with unique characteristics and properties, such as bright luminescence, high photostability, and strong biocompatibility. For example, modifying aptamers or antibodies on a fluorescent nanoprobe provides high selectivity and specificity for different objects to be tested. Fluorescence intensity, life, and other parameters of targets can be changed by different sensing mechanisms based on the unique structural and optical characteristics of fluorescent nanoprobes. What’s more, the detection of fluorescent nanoprobes is cost-saving, simple, and offers great advantages in rapid food detection. Sensing mechanisms of fluorescent nanoprobes were introduced in this paper, focusing on the application progress in pesticide residues, veterinary drug residues, heavy metals, microbes, mycotoxins, and other substances in food safety detection in recent years. A brief outlook for future development was provided as well.

## 1. Introduction

With the accelerating globalization of the food industry, the quality and safety of food have been drawing more and more attention from the public. Food quality includes nutritional value, functional characteristics, flavor, and the presence of contamination. Food safety problems [1] mainly include the contamination of pesticides [2], veterinary drugs [3], residues of heavy metals [4], microbes [5], mycotoxins, and other toxicants [6,7]. According to the physico–chemical properties of these pollutants, GC-MS [8,9], LC-MS [10,11,12], and HPLC [13] are applied by food analysis researchers. Despite the advantages of these methods, complex preprocessing, requirements for highly skilled inspectors, long testing times, the need for a large number of organic reagents, expensive precision instruments, and high costs limit the food safety analysis and application of some small-scale food enterprises with limited resources. Therefore, it is of great significance to develop efficient, convenient, sensitive, fast, and reliable new detection methods to ensure food safety and human health.

In current methods based on sensors, fluorescent sensors are relatively common analytical tools. Organic dyes, such as rhodamine, fluorescein, and anthocyanins, are the most commonly used fluorescent probes. However, most organic fluorophores under long light exposure will develop irreversible photobleaching, which results in short duration, low absorption, and many other shortcomings for detection. Some organic dyes also have strong phototoxicity. With the progress in the field of nanoscience, fluorescent nanoprobes with properties of brighter luminescence, higher photostability, and better biocompatibility are drawing more and more attention. Through surface modification, precise control of the size, shape, chemical composition, and surface chemistry of fluorescent nanoprobes, unique optical properties, and high performance are obtained [14]. Compared with commonly used fluorescent nanomaterials, the quantum dot is superiorly resistant to degradation, has long photostability, and has high brightness due to its inorganic properties. Owing to its nanosize, higher drug-carrying capacity is also ensured. The upconversion nanoparticles (UCNPs) have the unique phenomenon of anti-Stoxluminescence [15]. UCNPs containing Lanthanide ions (Ln^3+^) could be excited by multiple low-energy photons and then emit a single high-energy photon at a shorter wavelength. This process is called upconversion luminescence (UCL) [16]. Meanwhile, they have a small physical size, a larger sample penetration depth, a sharp emission bandwidth, a long life, high photostability, and low cytotoxicity. Moreover, their long-wavelength emission is relatively weakly absorbed by the biological samples, which avoids the interference of spontaneous fluorescence in the samples [17,18]. Gold nanoparticles (AuNPs) are particles with controlled size and morphology, good biocompatibility, a strong surface modifier, and the inherent properties of surface plasmon resonance (SPR). The color signals of AuNPs are generated based on the distance between the particles, which achieves a visual assessment of food quality [19,20]. Fluorescent nanomaterials could overcome the shortcomings of traditional detection methods. It shows a great application prospect in food testing, which meets the needs of rapidness, lower cost, and efficiency of modern food testing, such as rapid food detection and on-site risk screening, which are conducive to the timely and rapid assurance of food safety from the source.

Based on these excellent properties of fluorescent nanomaterials, new methods have been developed for food safety detection, which provide a reference for wider application in the field of food safety and provide new ideas for food safety detection in the future.

## 2. Fluorescent Nanoprobes

Fluorescence was discovered and reported by Frederick William Herschel in 1845. George Stokes described more detail of fluorescence in 1852 [21]. Fluorescence is the light emitted by the excited electron paired with the electron in the ground state in the excited singlet [22]. The analysis of different analytes could be realized by using different fluorescence parameters, such as fluorescence intensity, isotropy, lifetime, quantum yield, and quenching efficiency [23]. With the continuous development of fluorescence technology, many fluorescence-based detection methods are used in different fields, such as food safety, drug delivery, environmental monitoring, and biological imaging. The gradual maturity of nanotechnology also provides new opportunities for fluorescent sensors. When an analyte molecule or an ion interacts with the nanocrystalline surface of a fluorescent nanoprobe, the fluorescence parameters will be altered [24]. The mechanisms are mainly based on fluorescence resonance energy transfer (FRET), photon-induced electron transfer (PET), chemiluminescence energy transfer (CRET), chain aggregation, or conformational transformation. FRET is a phenomenon occurring between two close fluorophores. When the distance between two fluorophores is less than 10 nm, the donor fluorophore is excited. The donor fluorophore transfers energy to the receptor chromophore through non-radiative dipole-dipole coupling. Thus, the fluorescence intensity and excitation lifetime of the donor fluorophore decrease, while the emission intensity of the receptor fluorophore increases [25,26]. PET occurs when the fluorophore is excited and the electrons are transferred from the highest occupied molecular orbit (HOMO) to the lowest unoccupied molecular orbit (LUMO) and generate holes in HOMO. The electrons are transferred from HOMO of the donor fluorophore to LUMO of the receptor fluorophore due to the redox reaction. As a result, the HOMO of the receptor fluorophore is occupied, the receptor fluorophore will no longer be able to return to its ground state, and fluorescence changes [27].

CRET refers to the chemiluminescence of the donor light-emitting substance without requiring an external excitation light source. The energy transfers to and activates the excitation receptor [28,29]. Conformational transition mechanisms, such as aggregation-induced emission (AIE), aggregation-induced quenching (ACQ), and depolymerization-induced emission (DIE), manifest when the fluorophore conformation changes (twist, folding, etc.). The energy transmission also changes the luminescence of the fluorophore. By means of these mechanisms [30], fluorescent nanomaterials are applied to detect agricultural and veterinary drugs, heavy metals, microorganisms, and mycotoxins in food samples (Table 1).

## 3. Applications in Food Safety Detection

### 3.1. Pesticides

In the process of food production, pesticides play an important role in ensuring the production of crops, but food safety problems caused by pesticides draw public attention [55]. In order to reduce these problems, highly sensitive, rapid, and simple methods needed to be developed to detect pesticide residues.

Fluorescent nanoprobes have good optical properties and biocompatibility. Meanwhile, it can combine specifically with the target material, reduce the background interference, improve the sensitivity to some extent, save cost, and increase detection speed. The ratiometric fluorescent probes are made up of a response/indication signal and a reference signal. A stable detection system is constructed through the self-calibration of ratiometric fluorescent probes, so complex systems can be detected accurately. Nafiseh Fahimi-Kashani developed a ratiometric fluorescent probe by utilizing a yellow emissive cadmium telluride (CdTe), which is a quantum dot (QD), and a blue emissive carbon dot (BCD) to detect Methyl parathion (MP) [31]. This study self-assembles cetyltrimethylammonium ammonium bromide (CTAB) on the QD surface. Electron transfer between MP and CdTe quantum dots was promoted through strong hydrophobic interactions between MP and CTAB, and electrostatic interaction between MP and the probe was enhanced. Therefore, the fluorescence of CTAB-CdTe was quenched, and the intensity of BCD was maintained. The concentration of MP showed a good linear relationship with the fluorescence response intensity of the probe within 0.001–10 μg/mL, with an R^2^ of 0.995 and a LOD of 1.2 ng/L. When spiked levels of MP in rice, wheat flour, and tap water were 2, 3.5, and 5 μg/mL, respectively, recovery rates were within 98–102%, RSD < 3%. Meanwhile, the limit of detection (LOD) of MP in rice was 4.63 μg/mL. The results of the comparison experiment showed that this method was accurate and feasible. The ratiometric fluorescent probes used in this study were enzyme-free, widely applicable, highly selective, and sensitive. It had the potential to be applied to the rapid visual detection of MP coupled with smartphones in real-time detection. Other research groups have developed different ratio metric fluorescent nanoprobes, such as cadmium telluride quantum dots (cdTeqDs), blue-emitted nitrogen-doped carbon quantum dots (NCQDs), and red-emitted copper nanoclusters (CuNCs), rQD@PS@bCD [32,67], so that carbaryl, thiram, paraquat, and 2,4-Dichlorophenoxyacetic acid were quantified on-site rapidly.

The special luminescence properties of rare earth nanocrystalline materials, applied as upconversion nanoparticles (UCNPs), have attracted the attention of researchers. These nanoprobes improved the sensitivity to some extent and had the advantages of a narrow emission peak, large Stokes displacement, low toxicity, high quantum yield, good chemical stability, and non-autofluorescence [52]. Yirong Guo et al. [33] established a fluorescence immunoassay based on graphene oxide (GO) and UCNPs by FRET for the rapid detection of imidacloprid. This method had a good linear relationship within 0.08–50 ng/mL (correlation coefficient of 0.9934), and the limit of quantification (LOQ) was 0.08 ng/mL. Recoveries of imidacloprid in the matrices (water sample, cabbage, carrot, honey, and tea) were good; RSDs < 14.3%. Seven samples were detected to be positive in 15 honey samples, which were consistent with the results of UPLC-MS/MS. Due to their unique properties, UCNPs effectively eliminate the background interference of autofluorescence in samples, which improves detection sensitivity. Wei Sheng et al. [34] utilized hydrophilicNaYF4: Yb/Er UCNPs conjugated to the anti-atrazine antibody as the signal probe and polystyrene magnetic microspheres (PMMs) conjugated to the coating antigen as the capture probe. In this study, atrazine in sugarcane juice and river water samples was detected directly, with LODs of 2 ng/L and 20 ng/kg.

Aptamers are often used to modify nanofluorescent probes due to their good specificity. Based on the specific recognition ability of nucleic acid aptamers and amidine, Xia Lu et al. [35] developed a new nanofluorescent nucleic acid aptamer sensor to recognize acetamiprid by using RecJfexo nuclease to generate photo-induced electron transfer between β-CD-CD and 3’ end-marked ferrocene (Fc), which resulted in fluorescence quenching. Specific binding of aptamers to the acetamiprid ensured high selectivity. It also showed a good linear relationship within 5 nM–1.2 μM, with an R^2^ of 0.995, a LOD of 3 nM, and RSDs < 3.9. The spiked levels of acetamiprid in honey and orange juice were 5, 10, and 50 nM, with recovery rates ranging between 94.9 and 104.2% and 96.8 and 105.5%, respectively, and RSDs < 4.01%. Compared with other detection methods, this nanofluorescent nucleic acid aptamer sensor showed good sensitivity, simple sample preprocessing, easy preparation, was non-toxic, and was cost-saving. Rajni et al. [36] synthesized CdTe@CdS quantum dots, poly(N-(3-guanidinopropyl) methacrylamide) homopolymer (PGPMA), and malathion-specific aptamers for specific detection of malathion. As a result of the existence of malathion, the aptamer bound to malathion, and the quantum dot fluorescence was quenched. This method showed a good linear response within 0.01 nM–1 μM, with a LOD of 4 pM.

### 3.2. Veterinary Drugs

Currently, there are more than 200 kinds of veterinary drug residues regulated in food; they are mainly antibacterial, antiparasitic, and antiviral [68]. Veterinary drugs are used in animal husbandry to keep animals healthy. However, improper use may cause veterinary drug residues on the food chain, which result in directly toxic effects on the human body or potential harm to health [68]. Antibiotics are commonly used in veterinary drugs that may cause anti-microbial resistance (AMR). According to the estimation of the World Health Organization (WHO), 700,000 people die from AMR every year. If this situation continues, 10 million people may die from drug-resistant bacterial infections every year by 2050 [69].

As one of the broad-spectrum antibiotics, tetracyclines (TC) are widely used to treat bacterial infections. Based on Attapulgite (Atta), Lei Jia et al. [37] designed an Atta-Eu-FITC (fluorescein isothiocyanate) nanoprobe for the detection of tetracycline. In the absence of TC, a certain amount of coordinating water molecules around the europium ion quenched the fluorescence of Eu^3+^. So that the probe showed green fluorescence from FITC. In the presence of TC, the fluorescence intensity of FITC as the internal standard of fluorescence was unaffected, while the characteristic emission peak intensity of Eu ions at 593, 617, 652, and 695 nm gradually increased. So that the probe showed continuous fluorescence changes from green, yellow, orange, and finally red after the addition of TC. This method had good linear relations in the range of 0–25 μM, with a LOD of 19 nM. The fortified recoveries of TC in milk and honey were 104.8–105.9% and 102.8–105.1%, respectively, with RSDs of 4.4–4.8% and 2.8–3.3%. In view of the high selectivity and sensitivity of the probe, a wearable smart glove-type nanosensor for the analysis of TC on food surfaces was developed. TC residues on the surface of fruit samples were detected rapidly, visually, and qualitatively. In addition, with the help of applications for color capture and analysis on smartphones, an intelligent method for TC on field tests was provided.

Cephalosporins are a widely used class of antibiotics. Opas Bunkoed et al. [39] embedded graphene quantum dots and magnetic nanoparticles in molecularly embedded polymers (GQDs@Fe_3_O_4_/MIP) and developed a magnetic nanocomposite fluorescent probe to enrich and detect ceftazidime residues in milk. The probe has a good linear relationship between 0.10 and 10.0 μg/L, with a LOD and a LOQ of 0.05 and 0.17 μg/L, respectively. The recovery rate of ceftazidime in milk was 90.7–99.2%, and the RSD < 6%. The method used a light-sensor graphene quantum dot with good water dispersion, biocompatibility, chemical stability, low toxicity, high photo-stability, and a high surface volume ratio. These properties give the sensor high sensitivity and molecularly imprinted polymer specificity, which ensure the high selectivity of the nanocomposite probe. Meanwhile, magnetic nanoparticles realize the detection of small volumes of solution, maintain the advantage of high sensitivity of light sensors, and complement the selectivity disadvantage of quantitative detection of ceftazidime. Saeedeh Narimani et al. [41] developed a new high-fluorescent CD using a green, low-cost, and simple hydrothermal method without surface passivation or chemical reagents. Due to this probe, ceftriaxone (CTR) was detected based on the internal filtration effect. The fluorescence intensity decreased in the presence of CTR, with a good linear relationship within 9 × 10^10^ M–1.4 × 10^8^ M and a LOD of 4.4 × 10^10^ M. The recovery rates of CTR in human urine were within 99.3–103.0%, and the RSD was 3.3%. Ai-Yue Hao et al. [42] assembled red-emitting fluorescent CdTe QDs (r-QDs) on the surface of blue-emitting fluorescent CDs as a response probe and modified the synthetic probe with a cephalexin antibody. Meanwhile, a dual emission ratio fluorescent probe was designed and combined with smartphone applications into a portable detection device, which was utilized for the specificity and visibility detection of cephalexin. This method scaled well linearly in the range of 1–500 nM, with a LOD of 0.7 μM. The recovery rates of cephalexin in milk were 94.1–102.2%.

Apart from tetracyclines and cephalosporins, Ning Lian et al. [38] developed a sensor of Cys-CuNCs-Fe^3+^ for the detection of ofloxacin (OFL) and norfloxacin (NOR). This sensor was based on the strong electron affinity and ferromagnetism of Fe^3+^. The fluorescence intensity of Cys-CuNCs was changed due to the strong complexation of quinolones (QN) and Fe^3+^. OFL and NOR were in good linear relationships within 0.5–40 μM and 0.5–50 μM, and R^2^s were 0.9985 and 0.9987, respectively, with a LOD of 50 nM. The recovery rates of OFL and NOR in fresh human urine were 97.80–101.80% and 97.20–102.30%, respectively. Further purification of analytes was not needed in this method, which could overcome sample matrix interference and could be applied in drug quality control analysis of QN.

β-agonists improve the protein deposition of animals due to their good protein assimilation, so they are illegally used as animal growth promoters to improve the feed conversion rate and increase the growth of animal muscles [70]. However, β-agonists, when consumed by eating animal-derived food, cause headaches and nausea and even lead to death in serious cases.

Yalan Liu et al. [40] developed a CD sensor based on FRET between clenbuterol (CLB), and residues of CLB were measured rapidly due to the change in carbon dot fluorescence intensity. There was a good linear relationship in the concentration range of 8–200 nM in this study, with a LOD of 3 nM. The recovery rates of CLB in pork were 97.5–105.0% and the RSDs were 2.32–4.35%. Further functionalization of the nanoparticles used in this method was not needed. Therefore, the preparation of the sensor was simple and fast, which avoided tedious experimental steps and provided a new detection method for CLB.

### 3.3. Heavy Metals

The fluorescent nanoprobes in a ground-state or excited state bind to the target metal ions, which causes energy or charge/electron transfer. The fluorescence properties of the probe are thereby changed, which causes fluorescence enhancement or quenching [71]. Based on this principle, fluorescent nanoprobes are important materials for the development of simple and reliable metal-ion analytical methods.

Mercury in the environment is easily converted into methylmercury with higher toxicity, which accumulates in humans through the food chain and causes serious neurotoxic damage. Yanglei Yuan et al. [44] developed a simple two-step method to synthesize a tetraphenylethylene derivative based on a fluorescent probe, which is based on the aggregation-induced luminescence principle (AILP). As a result, mercury was detected selectively and reversibly in this study. In the presence of Hg^2+^, poor water-soluble complexes were generated with the probe, which had an AILP effect with an enhanced fluorescence response. Owing to the addition of S^−^ to the detection system, Hg^2+^ was removed from the complex of the probe, and the fluorescence response returned to a weak state for reversible detection. This method showed a good linear relationship within 15–45 μM, with a LOD of 45.4 nM. The recovery rate in tap water was 103.1, and the RSD was 1.1%. This method had high sensitivity and good selectivity. What’s more, its reversible detection of Hg^2+^ could be generalized to remove toxic Hg^2+^ from tap water or environmental samples.

Fe^3+^, one of the essential microelements in human nutrition, plays a very important role in many physiological and pathological processes, and the excess or deficiency of Fe^3+^ causes health problems [72]. Linli Hou et al. [45] developed 2,5-dihydroxyterephthalic acid (DOBDC)-Zn^2+^ MOFs (ZnMOF-74) to detect Fe^3+^. ZnMOF-74 was a sensor with good thermal stability, a high surface area, and excellent biocompatibility due to its unique pore structure. In aqueous solution, as a result of the special complexation reaction between Fe^3+^ and the phenolic hydroxyl group of DOBDC, the cation exchange between Fe^3+^ and Zn^2+^ occurred, and the skeleton collapsed. What’s more, a blue, weak-fluorescent iron (III)-phenol salt complex (Fe-DOBDC) formed, which led to an effective fluorescence quenching of ZnMOF-74. To detect Fe^3+^ under more acidic conditions, the linear relationship was good within 0.1–100 μM, with a LOD of 40 nM. The recovery rates of Fe^3+^ in river water samples were in the range of 104.1–108.9%, and those of Fe^3+^ in human serum samples were 101.5–103.5%. Due to its special structure, the sensor had fluorescent stability with a wide linear range and a low LOD, and Fe^3+^ could be detected visually and applied to the detection of Fe^3+^ in water samples.

Cobalt is an environmental poison that damages the human nervous system. Studies have shown that low doses of cobalt (significantly below the safety threshold) still induce neurodegenerative changes [73]. Shiwei Yang et al. [74] designed a nanofluorescent probe for detecting Co^2+^ by modifying Mn-doped ZnS quantum with b-Cyclodextrin (b-CD) in an acidic aqueous solution by a one-step method. The linear relationship in this method was within 1–10 μmol/L in actual water samples, with a LOD of 0.26 μmol/L.

Lead, one of the most toxic heavy metals, accumulates in humans for decades. The International Agency for Research on Cancer (IARC) classifies lead as a class 2B carcinogen, and even a trace amount of lead is considered unsafe [46]. Sardar Paydar et al. [75] changed the optical properties of the metal nonreactive carbon point (Cdot) by the modification of glutathione (GSH), using the modified Cdot as a highly sensitive and selective ratio-type probe for the detection of Pb^2+^. The sulfur lone pair electrons of glutathione inhibited the modified Cdot. The GSH-modified Cdot accumulated and produced a new emission peak at longer wavelengths in the presence of Pb^2+^. This method maintained a good linear relationship in the concentration range of 10–700 nM, with a LOD of 2.7 nM. In the actual sample detection, Pb^2+^ was not detected in tap water or rivers, but the recovery rates of Pb^2+^ in industrial wastewater were 98.0–103.1%, and the detection concentration (including dilution factor) was 13.45 ± 0.07 μM (2787 ± 16 ppb) (n = 5). The results showed that the probe was not affected by the matrix effect of river and industrial water samples, and Pb^2+^ could be detected reliably and efficiently in actual water samples.

The determination of various metal ions has become more and more necessary; however, the identification of single metal ions has been interfered with by other coexisting metal ions. Zhe Jiao et al. [43] utilized different quenching effects of copper, mercury, silver, and cadmium with manganese-doped ZnS quantum dots (Mn: ZnS QDs), which were modifications of N-Acetylcysteine, citric acid, mercaptopropionic acid, and triammonium-N-dithiocarboxyiminodiacetate and A sensor array presenting different fluorescence responses to different metal ions was designed. This method showed good linear relationships for all analytes (R^2^ ≥ 0.96), and LODs were between 0.3 and 2.7 pg/mL. Six binary mixtures (Ag/Cd, Cu/Hg, Cu/Ag, Hg/Cd, Ag/Hg, and Cu/Cd) were identified, and four metal ions (copper, mercury, silver, and cadmium) were classified. The accuracy of metal ion recognitions obtained in actual water samples was 100%. The sensor array, simply synthesized by modifying ZnS quantum dots with different ligands, overcame the identification of other single metal ions interfered with by coexisting metal ions. Furthermore, it was an alternative to easy-to-prepare and low-cost noble metal nanoclusters or enzyme-based approaches to traditional methods.

### 3.4. Microorganisms

Pathogenic bacterial infections and related foodborne diseases caused by some pathogenic microorganisms in food products cause global public health problems and economic losses [76]. Bacteria are detected due to the self-functionalization of fluorescent nanoprobes or unique microbial properties (such as bacterial copper stabilization mechanisms).

*Escherichia coli* (*E. coli*) poses a serious threat to human and animal health and a huge economic loss as well [77]. Meanwhile, *E. coli* is a widely studied bacterium that is commonly used as an indicator of fecal contamination [78]. Yang Song et al. [47] used a one-step redox reaction to synthesize papain-modified gold nanoclusters (papain@AuNCs), which were modified with an aptamer to capture *Escherichia coli* O157: H7. Afterward, a complex of bacteria@aptamers@papain@AuNCs with significantly different signals from other common food-borne pathogens formed, which had higher oxidase-like activity and better resistance to complex dairy substrates. This study detected milk samples from different sterilization methods (UHT sterilized, pasteurized, and raw milk). This method, which is less irritating, toxic, and corrosive, had a good linear relationship within 10^2^–10^6^ CFU/mL, R^2^ ≥ 0.97. LODs were 5.6 × 10^2^ CFU/mL, 5 × 10^2^ CFU/mL, and 4.9102 CFU/mL. What’s more, compared with the traditional BSA@AuNCs colorimetric method, the fluorescent signal was stronger at 652 nm. In addition, the probe, which was specific, rapid, and safe, avoided dangerous and complicated experimental steps.

*Salmonella typhimurium* (*S. typhimurium*) is one of the main pathogens causing acute gastroenteritis. Qingmei Chen et al. [50] modified bovine serum albumin-stabilized gold nanoclusters (aptamers@BSA-AuNCs) to enhance bacterial peroxidase-like activity, which promoted BSA-AuNCs generation and blue charge-transfer complexes as the principle established for *S. typhimurium*. The selective detection method had a good linear relationship within 101–106 CFU/mL, with a LOD as low as 1 CFU/mL. The recovery rates in eggshells and egg white were 92.4–110%. The method, observed by the naked eye with high sensitivity, required neither sophisticated instruments nor complex sample pretreatments. As a result, it had a good application prospect in food quality testing.

Based on the metal coordination interaction of (3-thiol-propyl) trimethoxysilane, magnetic γ-Fe_2_O_3_, and fluorescent quantum dots, Linyao Li et al. [48] self-assembled FMNP probes with different magnetic responses and excellent fluorescent quality. The apt-FMNPs nanoprobe was later modified with aptamers specifically for E. coli O157:H7 and S. typhimurium. Due to the differential magnetic response strength of the pathogen@nanoprobe complex at the same external magnetic field, the bacteria were captured and detected at different time points with magnetic adsorption in the milk. The recovery rates were 84.9–95.9% and 87.6–97.7%, respectively, with RSDs of 1.48–5.91% and 1.1–4.3% and LODs of 150 CFU/mL and 100 CFU/mL.

*Campylobacter jejuni* (*C. jejuni*) is the main pathogen of severe bacterial gastroenteritis, which mainly enters the human body, especially through poultry food. Zahra Dehghani et al. [51] designed and synthesized an ON/OFF fluorescent biosensor by using the principle that *C. jejuni* controlled the distance between graphene oxide (GO) and graphene quantum dot (GQD), which resulted in fluorescence resonance energy transfer (FRET) and changes in fluorescent response. This method possessed a good linear relationship within 10–106 CFU/mL, with a LOD up to 10 CFU/mL. The recoveries of C. jejunum in poultry liver were between 93.3 and 104.1%. This method was more sensitive and less time-consuming than SPR, ELISA, and electrochemical methods, which could be tested within 1.5 h, and different pathogens could be separated in food samples.

Based on the bacterial copper homeostasis mechanism, Li Fu et al. [49] designed a selective and antibody-free ratio-type fluorescent probe in order to detect gram-negative bacteria quantitatively. The method had a good linear relationship within 103–107 CFU/mL, with a R^2^ of 0.943. The LODs of three gram-negative bacteria (*E. coli*, *P. aeruginosa*, and *S. typhimurium*) were 150, 112, and 792 CFU/mL, respectively. The recovery rates in eggshell and Chinese cabbage were 93.9–109%, with RSDs < 5.4%. This method was easy to operate and performed better than other sensors in terms of response time.

### 3.5. Mycotoxins

Fungal toxins, secondary metabolites of fungi, are produced during fungi growth in food products under specific conditions. The hazards of mycotoxins are their colorless and tasteless qualities, and they remain viable even after inactivation, so they exist at all stages of production and processing. Mycotoxins include aflatoxins (AFs), ochratoxin A (OTA), fumonisin B (FB), deoxynivalenol (DON), T-2 toxin, zearalenone (ZEN), and so on. The most toxic ones [56,79] are AF and OT. AFB_1_ and AFB_2_ can be converted to hydroxylated AFM_1_ and AFM_2_ in lactating cows after ingestion of contaminated feed. OTA with hepatorenal toxicity is mainly detected in cereals, coffee, wine, grape juice, and dried fruits [80]. FB, with oxidative stress and mitochondrial dysfunction altering apoptosis and proliferation, was found both in maize and maize by-products around the world [81]. DON, which has immune toxicity, oxidative damage, haptoral and renal toxicity, and reproductive toxicity, was mainly detected in barley grains, chicken, eggs, feed, and wheat [82,83,84]. T-2 toxin, which causes various cardiovascular diseases, was detected in drinking water and Chinese herbal medicine [85].

Since most fungi produce more than one mycotoxin, the development of new methods that meet the requirements of high sensitivity and multiple assays of mycotoxins is essential for food quality and safety. Imran Mahmood Khan et al. [86] combined the bicolor gold nanoclusters (AuNCs) with L-proline and BSA and then used the aptamer-modified Lp-AuNC and BSA-AuNC as donors and tungsten disulfide WS_2_ as an acceptor. In the process of FRET, AFB_1_ and ZEN were detected due to fluorescence intensity changes. When there was no AFB_1_ or ZEN, the fluorescence of AFB_1_ apt-Lp-AuNC or ZEN apt-BSA-AuNC was quenched by WS_2_. In the presence of AFB_1_ or ZEN, the toxin had a high affinity for the aptamer, the effect of FRET within AuNC and WS_2_ was blocked, and the fluorescence intensity was enhanced. The method had a good linear relationship within 0.005–100 nm, with LODs of AFB_1_ and ZEN of 0.3437 and 0.5384 pg/mL, respectively. The recoveries of AFB_1_ and ZEN in maize were 98–118.73% and 94.36–110.4%, respectively. Compared with the conventional FRET-based method, there are several advantages to this method, which has a single excitation wavelength and a high fluorescence quenching ability of WS_2_. Moreover, AFB_1_ and ZEN could be detected simultaneously. So a simple, efficient, economical, and FRET-based probe was developed, through which multiple toxins were separated and simultaneously identified in this study.

Yi Yang et al. [53] designed a high-throughput photonic crystal microsphere (PHCM) suspension array for the detection of multiple mycotoxins in grains by using aptamer fluorescence signal recovery technology. The concentration of the mycotoxin bound to its aptamer was indicated by the intensity of fluorescence recovery. The dynamic linear detection range of AFB_1_ exceeded 0.1 pg/mL-0.1 ng/mL. OTA and FB_1_ showed good linear relationships in the range of 0.1–10 ng/mL, and LODs for AFB_1_, OTA, and FB_1_ were below 16 pg/mL. Spiked levels in wheat, corn, and rice samples were at 0.001, 0.01, and 0.1 ng/mL for AFB_1_ and OTA, and 0.1, 1.0, and 10 ng/mL for FB_1_, while the recovery rates of them in different matrices were 79.88–108.84% (RSDs < 7.7%), 71.20–113.19% (RSDs < 9.1%), and 76.99–105.96% (RSDs < 5.4%), respectively. Despite some recovery rates being lower than 80%, most of them are more than 80% for AFB_1_ and FB_1_, and all of them were more than 70%. This method had high sensitivity to more than one type of mycotoxins and overcame the difficulties of simultaneous high-throughput screening of multiple mycotoxins and small-volume detection.

Quansheng Chen et al. [54] used antigen-modified magnetic nanoparticles (MNPs) as a sensor probe. UCNPs whose functionalization of antibodies was improved as a signal probe were developed as a dual sensing method for magnetically induced separation and specific formation of antibody-target complexes for the detection of AFB_1_ and deoxynivalenol (DON). The linear relationships between AFB_1_ and DON were in a wide range of 0.001–0.1 ng/mL. The recovery rates of AFB_1_ and DON in peanut oil were within 90.1–113.4% and 94.07–101.69%, respectively. This improved fluorescent probe showed stronger fluorescence properties and higher stability for storage.

María Celeste Rodríguez et al. [56] published a novel technique for grain mycotoxin quantification analysis based on fluorescence detection and stoichiometric models in order to overcome the shortcomings of traditional quantitative analysis of mycotoxins. This method showed good linear relationships between the aflatoxins (AFS) and OTA below 75.00 μg/L and 265.00 μg/L; the LODs of AFS and OTA in sorghum grains were 0.53 and 0.73 μg/L, respectively.

### 3.6. Others

Some food additives as well as toxins produced in the processes of production, transportation, and storage are harmful to the health of consumers. There are a large number of analytical methods for these substances in the food safety field, in which the fluorescent nanoprobe plays a unique role in the detection (Table 1).

Ascorbic acid (AA), a water-soluble vitamin, exists in fresh fruits and vegetables. AA is necessary to regulate human physiological function, so it is used as a nutritional supplement or antioxidant in food additives. Excess and deficiency of AA harm human health. Xuan Wang et al. [57] used AA to reduce Cu^2+^ into Cu^+^, which made Gu^2+^, Cu^+^, and Cu out of balance. And stable nanoparticles called CuNCs were produced due to the accumulation of Cu in dsDNA, so a novel method for rapid and label-free fluorescence detection of ascorbic acid based on the in situ formation of copper nanoclusters was developed. The LOD in this method was 41.94 μM, while recovery rates of AA spiked in human serum were within 92.19–103.09%, and the RSDs were below 4.4%. Because the CuNCs are formed in situ, there is no interference from the background or other substances, and fluorescent DNA-CuNCs can be formed in a few minutes, which provides a fast and reliable new path for the detection of AA. Xiemin Yan et al. [58] synthesized glutathione template-based GSH-AuNCs due to a chemical reduction method. In this method, the quencher OH^−^ was formed by the Fenton reaction between Fe^2+^ and hydrogen peroxide. Au in GSH-AuNCs was oxidized by OH^−^ in the presence of AA, and the fluorescence intensity recovered. The method had a good linear relationship within 350–700 μM with a LOD of 200 μM.

Xueliang Liu at el. [59] used polyvinylpyrrolidenone (PVP) as a reductant and stabilizer and synthesized PVP-stabilized Pt nanoclusters (PVP-Pt NCs) in a simple and cost-saving way, in which non-harmful chemical reagents were used as well. This method had a good linear relationship within 100–500 μM, with a LOD of 1.17 μM. The recovery rates of AA in vitamin C tablets and lemon soda drinks were in ranges of 98.72–101.37% and 97.39–97.85%, with RSDs of 1.16–3.44% and 2.61–3.17%, respectively.

Although the fluorescence properties of quantum dots have been utilized to detect ascorbic acid, previous reports have focused on fluorescence intensity changes (on or off). The stability of fluorescence intensity is not good enough and is easily affected by instrumental and environmental conditions. Therefore, Jian Zhu et al. [60] developed an AA detection method based on the wavelength offset of the fluorescence peak of CdTe. Compared with the fluorescence intensity, the wavelength offset of the fluorescence peak was of better accuracy and avoided the shortcoming of poor stability of the fluorescence intensity. AA induces a redshift in the fluorescence emission of CdTe, and this AA-dependent fluorescence emission redshift could be applied to detect and quantify AA. The method showed a good linear relationship in the range of 10–250 μmol/L, with a LOD of 1.3 μmol/L. The recoveries of AA at two spiked levels in commercial vitamin C tablets were 103.1%, 102.9%, and RSD < 7%.

Melamine is a nitrogen-containing organic compound that is illegally added to dairy products to raise the apparent protein levels determined by the Keldal method because of its low cost and high nitrogen content of 66%. Despite its low toxicity, melamine in high concentrations may lead to kidney disease and infant death. Melamine also forms an insoluble melamine-cyanate complex, resulting in kidney stones and renal failure [87]. Therefore, selective and quantitative detection of melamine is important for applications in public health and food safety supervision. XinYue et al. [61] developed a new method of melamine detection in lateral flow assays (LFA) based on the AIE of AuNCs. ATT-AuNCs emitted aggregation-induced fluorescence during detection, with a good linear relationship in the range of 1–100 μM and a LOD of 217 nM. The recovery rates of melamine in milk and infant formula were within 91.6–101.5%. This method maintained the advantage of low background interference as a result of the good separative capacity of LFA. Meanwhile, the use of aggregation-induced fluorescence recognition, avoiding the use of antibodies, aptamers, and other elements, reduced the cost.

Fei Qu et al. [62] and Gopi Kalaiyarasan et al. [63] used glutathione to design ratiometric-type fluorescent probes of CD and glutathione-stabilized gold nanoclusters (CNDs/GSH@Aunc), respectively. The linear relationships were good within 0.1–30 µM and 100 M–5 mM, with LODs of 29.3 nM and 28.2 µM, respectively, and both methods showed good recoveries in milk and infant formula.

The toxin may be produced in food production processes, such as tyramine and nitrite in fermented food and marinated meat. Intake of too much tyramine is harmful to human health and causes migraines or hypertension, while nitrite is prohibited on the list of 2A carcinogens.

Bisphenol A (BPA) exists in mineral water bottles or food packaging so that it can be taken by humans through the food chain, which affects reproductive, immune, nervous, and other biological functions. Qinghua Wang et al. [64] published a fluorescence detection method for tyramine based on a novel optical sensing material, GraQDs@MIPs, by combining hydrophobic CdSe/ZnS with graphite due to the reverse-phase microemulsion. This method had a good linear relationship in the range of 0.07–12 mg/L, with a LOD of 0.021 mg/L. The recovery rates in rice wine were between 90.00% and 103.75%. Wenting Li et al. [65] designed a ratio fluorescence sensor to detect nitrite in sausages based on FRET between MoS_2_ and AuNC. A good linear relationship was shown within 0.5–20 mg/L in this method, with a LOD of 0.67 nM. Based on the fluorescence color change of the sensor at different nitrite concentrations, visual detection of nitrite in the range of 1.0–20 mg/L was feasible thanks to the microfluidic chip of the smartphone. The recovery rates of nitrite in the sausage were 102.6–110.8%.

Wei Sheng et al. [66] applied a quantum-dot-based immune-chromatographic assay (QICA) as a fluorescent donor and a fluorescence quenching immune-chromatographic assay (FQICA) as an energy receptor to detect BPA based on the FRET effect. LODs of QICA and FQICA in distillate spirit (42%, *v*/*v*), cabbage, grass carp, and river water were 10 and 4 μg/kg (μg/L), respectively.

Food quality control is not only about the detection and supervision of toxic substances but also the judgment of other indicators of food, such as shelf life and the maturity of vegetables. Hengye Chen et al. [88] developed a sensor array of acid-sensitive cadmium telluride quantum dots (CdTe-TGA QDs) combined with stoichiometry that measured 32 kinds of Chinese traditional cereal vinegar (CTCV) rapidly, accurately, and sensitively. This method mainly identified organic acid and described, the aging time of CTCV based on melanoidins, since organic acid enhanced the ET and FRET of QDs, so that fluorescence was quenched. This method detected organic acids in a range of 0.5–5 mmol/L. No organic dyes were used in this study, which saved costs. Many other foods rich in organic acids can be detected to identify organic acids, such as the shelf life of yogurt, the maturity of fruits, and the brewing time of pickles.

Ou Hu et al. [89] found that flavonoids, catechins, and amino acids in different green teas quenched the fluorescence of QDs to some extent. A fast, accurate, and sensitive combination of NAC-capped ZnCdSe-CdTeQDs and stoichiometry was established for measuring 53 kinds of green tea. The recognition rate of this method was 100% in large-class-number classification (LCNC). Therefore, it is promising for applications in other herbal medicine, food, biometrics, and drug quality control.

## 4. Conclusions

This paper summarizes recent research based on fluorescent nanoprobes in the field of food quality and safety. Different fluorescent nanoprobes functionalized with compatible aptamers or antibodies were researched for different targets to enhance specificity and ensure high selectivity in complex matrixes. Some researchers also used sensing mechanisms (FRET, RET, etc.) to design the fluorescent nanoprobes, such as control of the distance from the targets, so that sensitivity was improved.

In situ formation of fluorescence nanomaterials also had great application prospects, not only because the interference of background was reduced but also because the detections were accelerated, especially DNA-CuNCs [57], which could be generated in situ in only a few minutes. What’s more, on-site rapid detection methods were also developed to quantify contaminants in food. Furthermore, smartphone applications were combined to realize field visual detection. Lei Jia [37] developed a visual analysis of the smart gloves, which implemented on-site rapid identification and intelligent detection.

In short, the application of fluorescent nanoprobes has made new achievements in the development of food safety detection methods, in which not only different sources and types of pollutants were detected and different indicators of food quality were evaluated, but also optimization and innovation in the detection performance were achieved. In the future, fluorescent nanoprobes will have broader applications in the field of food safety detection, which plays an important role in the supervision and management of food safety.

## Figures and Tables

**Table 1:** Summary of food safety detection for various targets based on nanoprobes.

Food Safety Detection	Target	Sample	Response Mechanism	Modified Elements	Limit of Detection	Reference
Pesticides	parathion-methyl	Rice, wheat flour	ET (electron transfer), Quantum confinement effect	cetyltrimethylammonium bromide	1.2 ng/mL, 3.3 ng/mL, 0.02 μmol/L	[31]
	Carbaryl	Apple	Quantum confinement effect	N/A	0.12 ng/mL	[32]
	Imidacloprid	Water, cabbage, carrot, honey, tea	FRET	antigen and antibody against imidacloprid	0.08 ng/mL	[33]
	Atrazine	River water, sugarcane juice, corn, rice	upconversion	anti-atrazine antibody	0.002 μg/L, 0.002 μg/L, 0.02 μg/kg, 0.02 μg/kg	[34]
	Acetamiprid	Honey, orange juice	ET	Aptamer	3 nmol/L	[35]
	Malathion	Water, orange juice	Quantum confinement effect	Aptamer	4 pM	[36]
Veterinary drugs	Tetracycline	Milk, honey, lake water, tap-water	Fluorescence change	3-aminopropyltriethoxysilane	19 nmol/L	[37]
	Ofloxacin	Human urine	ET	Fe^3+^	50 nmol/L	[38]
	Norfloxacin	Human urine,	ET, Antenna effect, Conformational change	Fe^3+^	50 nmol/L	[38]
	Ceftazidime	Milk	ET	Fe_3_O_4_, MIP	0.05 μg/L	[39]
	Clenbuterol Hydrochloride	Pork	FRET	N/A	3 nmol/L	[40]
	Cefatriaxone	Human urine	IFE (internal filtration effect)	N/A	4.4 × 10^10^ mol/L	[41]
	Cephalexin	Milk	Fluorescence change	Cephalexin antibody	0.7 μmol/L	[42]
Heavy metals	Ag^+^	Water	Fluorescence change	mercaptopropionic acid	0.3–2.7 pg/mL	[43]
	Hg^2+^	Water	AIE	citric acid	0.3–2.7 pg/mL, 45.4 nmol/L	[43,44]
	Cd^2+^	Water	Fluorescence change	triammonium-N-dithiocarboxyiminodiacetate	0.3–2.7 pg/mL	[43]
	Fe^3+^	Water	Conformational change	2,5-dihydroxyterephthalic acid	40 nmol/L	[45]
	Co^2+^	Water	FRET	β-Cyclodextrin	0.26 μmol/L	[46]
Microorganisms	*Escherichia coli* O157:H7	Milk with different sterilization methods (UHT sterilized, pasteurized, and raw milk)	Fluorescence change	Aptamer	5.6 × 10^2^ CFU/mL, 5 × 10^2^ CFU/mL, 4.9 × 10^2^ CFU/mL	[47]
	*Escherichia coli*	Eggshell, chinese cabbage, milk	Fluorescence change	Bovine serum albumin, Aptamer	150 CFU/mL, 100 CFU/mL	[48,49]
	*P. aeruginosa*	Eggshell, chinese cabbage	Fluorescence change	Bovine serum albumin	112 CFU/mL	[49]
	*S. typhimurium*	Eggshell, chinese cabbage, milk, eggshell, egg white	Fluorescence change	Bovine serum albumin, Aptamer	792 CFU/mL, 150 CFU/mL, 1 CFU/mL	[48,49,50]
	*C. jejuni*	Poultry liver sample	FRET	Poly clonal antibody	10 CFU/mL	[51]
Mycotoxins	AflatoxinB1	Corn, cereal, peanut oil, lotus seeds	FRET	Aptamer	0.3437 pg/mL, 15.96 fg/mL, 0.001 ng/mL, 1 ng/mL	[52,53,54]
	Zearalenone	Corn	FRET	Aptamer	0.5384 pg/mL	[28]
	Ochratoxin A	Cereal		Aptamer	3.96 fg/mL, 0.73 μg/L	[53,55]
	Fumonisin B1	Cereal		Aptamer	11.04 pg/mL	[53]
	Deoxynivalenol	Peanut oil		Antibody, antigen	0.001 ng/mL	[54]
	Aflatoxins	Sorghum grains			0.53 μg/L	[56]
Others	Ascorbic acid	Commercial vitamin C tablets, vitamin C effervescent tablet, lemon soda		Glutathione, Polyvinylpyrrolidone	1.3 μmol/L, 41.9 μmol/L, 200 μmol/L, 1.17 μmol/L	[57,58,59,60]
	Melamine	Milk, baby formulas spiked, raw milk, cow milk, infant formulas	AIE	6-Aza-2-thiothymine, glutathione	217 nmol/L, 29.3 nmol/L, 28.2 μmol/L	[61,62,63]
	Tyramine	Rice wine			0.021 mg/mL	[64]
	Nitrite	Sausage	FRET	AuNCs	0.67 nmol/L	[65]
	Bisphenol A	Cabbage, grass carp, river water	FRET	Antibody	4 μg/L	[66]

## Data Availability

Data sharing not applicable.
